# Unmasking the Hidden Danger: A Decade-Long Systematic Review of Case–Control Studies on Single Occupational Risks and Prostate Cancer

**DOI:** 10.3390/life13091820

**Published:** 2023-08-28

**Authors:** Caterina Ledda, Massimo Bracci, Alba Spadafora, Giuseppe Motta, Giuseppe Smecca, Dolores Catelan, Venerando Rapisarda

**Affiliations:** 1 Occupational Medicine, Department of Clinical and Experimental Medicine, University of Catania, 95124 Catania, Italy; venerando.rapisarda@unict.it; 2 Occupational Medicine, Department of Clinical and Molecular Sciences, Polytechnic University of Marche, 60121 Ancona, Italy; m.bracci@univpm.it; 3 Occupational Health and Safety Unit, Provincial Health Agency of Siracusa, 96100 Siracusa, Italy; alba.spadafora@asp.sr.it; 4 Occupational Medicine Unit, “Garibaldi” Hospital of Catania, 95123 Catania, Italy; giusmotta@gmail.com; 5 Prevention and Protection Unit, Provincial Health Agency of Ragusa, 97100 Ragusa, Italy; giuseppe.smecca@asp.rg.it; 6 Unit of Biostatistics, Epidemiology and Public Health, Department of Cardiac, Thoracic, Vascular Sciences and Public Health, University of Padova, 35131 Padova, Italy; dolores.catelan@ubep.unipd.it

**Keywords:** prostate cancer, occupational exposure, risk factors, systematic review, firefighters, night shift work, physical activity, chemical exposure, total worker health, predictive, preventive, personalized, participatory (4P) medicine

## Abstract

The present systematic review addresses the influence of occupational exposures on prostate cancer risk. Eleven studies were analyzed for a range of occupational exposures, including but not limited to firefighting, physical activity, night shift work, chemical exposure, and solar ultraviolet radiation. The results of the review reveal that firefighters exposed to harmful substances, individuals engaged in physically strenuous work, and workers with chronic night shift routines showed an increased likelihood of developing prostate cancer. Moreover, the review identified an increased risk associated with exposure to certain chemicals, including alkylphenolic compounds and benzene-related substances. The evidence underscores the importance of considering the cumulative effect of multiple risk factors in a comprehensive risk assessment. However, the conclusions indicate the necessity for further research to deepen these relationships and develop more effective strategies for the prevention of prostate cancer.

## 1. Introduction

Prostate cancer (PCa) is the second most common cancer among men worldwide and the fifth leading cause of cancer-related death [1]. The incidence of PCa varies geographically, with the highest rates found in developed countries such as the United States, Western Europe, and Australia [1,2].

The introduction of the prostate-specific antigen (PSA) test in the late 1980s led to a substantial increase in the detection of early-stage PCa cases [3]. However, concerns have been raised about the potential overdiagnosis and overtreatment of indolent tumors that may not pose a significant threat to a patient’s health [4]. As a result, guidelines for PCa screening have evolved over time, with a greater emphasis on shared decision making between patients and healthcare providers [5]. Overall, the 5-year survival rate for PCa is high, at around 98% [6]. However, survival rates can vary depending on the stage at diagnosis and other factors, such as age, race, and overall health [6].

The risk of developing PCa increases with age, particularly after the age of 50, with the majority of cases diagnosed in men over 65 years of age [6]. The incidence of PCa varies among racial and ethnic groups; African-American men and Caribbean men of African descent have a higher risk compared to white men, while Asian-American and Hispanic/Latino men have a lower risk [6]. Family history plays a significant role in the development of prostate cancer; men with a father or brother who had PCa are more than twice as likely to develop the disease [7]. This risk increases if several family members have been affected, particularly if they were diagnosed at a young age [8]. Genetic factors also contribute to risk, with several gene mutations linked to an increased risk of prostate cancer, including BRCA1, BRCA2, and HOXB13 [9,10,11]. Dietary habits have been associated with PCa risk, with a diet high in red and processed meats and dairy products and low in fruits and vegetables potentially increasing risk [12,13]. Obesity is another factor, as men who are overweight or obese have a slightly higher risk of developing prostate cancer, with the association being stronger for more aggressive forms of the disease [14]. Chronic inflammation of the prostate (prostatitis) may also increase the risk of PCa [15]. Hormone levels, such as higher levels of testosterone and insulin-like growth factor 1 (IGF-1), have been linked to an increased risk of PCa [16,17]. Lastly, environmental factors, including exposure to certain chemicals like pesticides and herbicides, may be associated with an increased risk of PCa [18,19].

Occupational exposure to certain chemicals, substances, and work environments may contribute to an increased risk of prostate cancer. Several studies have investigated the link between various occupations and PCa risk, with varying degrees of evidence. For instance, agricultural workers, particularly farmers and pesticide applicators, may have a higher risk of PCa due to their exposure to pesticides and herbicides [18]. A meta-analysis by Van Maele-Fabry et al. [19] reported a weak but significant association between pesticide exposure and PCa risk. Workers exposed to polycyclic aromatic hydrocarbons (PAHs), such as firefighters, aluminum smelter workers, and coke oven workers, may have an increased risk of PCa [20,21,22,23]. PAHs are generated during the incomplete combustion of organic materials and are known carcinogens. Men engaged in shift work, particularly those working night shifts, may also have an increased risk of PCa [24,25]. This may be due to disruptions in the circadian rhythm and the suppression of melatonin production, which has been hypothesized to have a protective effect against cancer development [26].

The International Agency for Research on Cancer (IARC) has already recognized several agents with limited evidence in humans as being potentially carcinogenic with respect to prostate cancer. These agents include androgenic (anabolic) steroids, arsenic and inorganic arsenic compounds, cadmium and cadmium compounds, occupational exposure as a firefighter, malathion, night shift work, consumption of red meat, the rubber manufacturing industry, thorium-232 and its decay products, and X and gamma radiation [27,28,29,30,31,32,33].

However, the evidence at hand, while compelling, necessitates further examination and substantiation through additional research to firmly establish a clear link between occupational exposure and PCa risk. The interplay between occupational risk factors and PCa is a multifaceted issue that is likely influenced by a host of other factors, including genetic predisposition, lifestyle elements, and the duration and intensity of exposure.

Therefore, additional research is paramount to not only illuminate these intricate connections but also to drive the creation and implementation of effective prevention strategies. It is critical to continue to deepen our understanding, and this urgency underscores the value and need for thorough, systematic reviews such as this one.

## 2. Materials and Methods

### 2.1. Search Strategy

The primary objective of this review was to evaluate case–control studies concerning workers with a confirmed diagnosis of PCa.

This review is exclusively centered on case–control studies due to their distinctive advantages when investigating rare outcomes, such as specific cancers linked to occupational factors. These studies offer an in-depth analysis of potential risk factors among individuals diagnosed with the disease, facilitating a more targeted investigation of occupational risks in relation to PCa. Moreover, in terms of both time and cost, case–control studies are typically more efficient compared to cohort studies.

A comprehensive search was performed in four electronic databases: PubMed, Web of Science, Scopus, and the Cochrane Library. The search strategy combined medical subject headings (MeSH) and relevant keywords. The search string comprised terms such as “occupational risk factors”, “work exposure”, “employment”, “prostate cancer”, and “neoplasms”. Additional filters were added to refine the search, which included human studies, English language, and peer-reviewed articles [34].

### 2.2. Selection Criteria

Included studies had to meet the following criteria: (1) original research articles, (2) case–control studies focusing on specific occupational risk factors and prostate cancer, and (3) studies published in English. Exclusion criteria were (1) reviews, case reports, commentaries, conference papers, and editorials, as well as (2) studies that did not provide sufficient data for analysis. The study selection followed the PRISMA guidelines [35].

In this systematic review, the definition of a ‘case’ was directly sourced from the original authors of each included study. These researchers specifically considered patients diagnosed with PCa based on their established criteria and methodologies.

### 2.3. Data Extraction

Data were extracted by two independent reviewers using a predefined extraction form. The data included authors, year of publication, study design, sample size, type of occupational exposure, assessment technique, and main findings. Disagreements between the reviewers were resolved through discussion or consultation with a third reviewer.

## 3. Results

### 3.1. Characteristics of Eligible Studies

**Figure 1:**
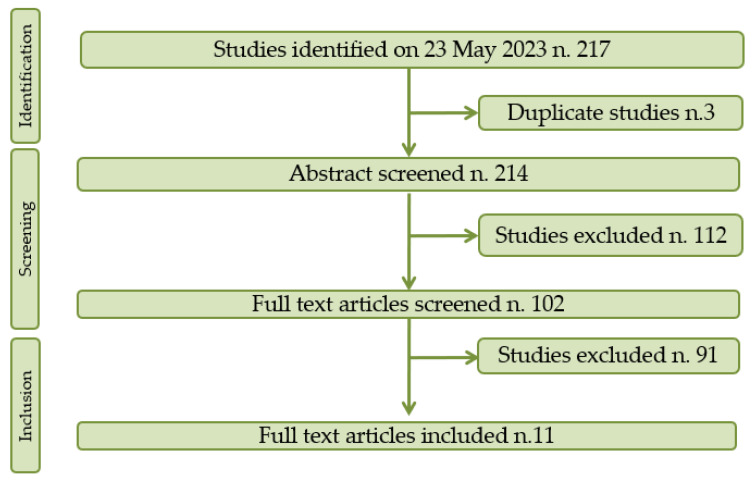
PRISMA flow chart of the selection process.

Following a search of the relevant databases, 214 documents were identified, of which 112 were excluded after review of the title and abstract and 91 of which were excluded after evaluation of the manuscript. Ultimately, eleven studies satisfied the inclusion criteria and were included in the systematic review. A flow chart depicting the study selection process is shown in Figure 1.

### 3.2. Results of Eligible Studies

The data presented in the Table 1 concern various studies conducted in different countries, investigating the possible association between occupational exposure to different risk factors and PCa.

A plurality of studies were conducted in Canada (5 of 11 studies, which equates to 45.5%) [36,37,38,42,43]. The United States contributed two studies, making up 18.2% of reviewed studies [40,44]. These studies primarily targeted firefighters, indicating an interest in the risks faced by this occupational group within the American context.

Spain also contributed two studies, constituting 18.2% of the total [25,41]. These studies investigated various risk factors, including night shift work and exposure to alkylphenolic compounds, highlighting diverse areas of interest within the Spanish research community.

Single studies were conducted in Australia and France, each representing 9.1% of the total studies. They explored various risk factors, from physical activity at work in Australia to different patterns of night shift work in France [39,45].

Regarding the study size, there was significant variation, with smaller studies consisting of as few as 114 cases [43] and larger studies including up to 1933 cases [37]. Similarly, the control groups varied widely in size, from 400 in one of the Canadian studies [37] to a considerable 63,912 [40] in one of the U.S. studies examining firefighters.

A diverse range of occupations and risk factors was studied, including perceived work stress, physical activity at work, exposure to certain chemicals, firefighting, night shift work, solar ultraviolet radiation, and farming and firefighting activities. Across the 11 studies under review, a variety of methods was employed to assess the outcome measure—specifically, the incidence of prostate cancer. In several studies, such as those conducted by Blanc-Lapierre et al. in 2017 and Doolan et al. in 2014, data were collected via questionnaires and/or interviews [36,37,39,43,45]. A study conducted by Blanc-Lapierre et al. in 2018 utilized the Canadian occupational classification [38]. The studies by Lee et al. in 2020 and Tsai et al. in 2015, which focused on firefighters, employed a firefighter registry to track the incidence of PCa [40,44]. The research conducted by Papantoniou et al. in 2015 took a different approach, using data nested from the MCC-Spain study, a comprehensive population-based case–control study [25]. This research project collected a wide range of data on various health outcomes, including prostate cancer and associated risk factors, enabling researchers to draw from a vast pool of pre-existing data. Meanwhile, the studies conducted by Peremiquel-Trillas et al. in 2019 and Peters et al. in 2016 utilized a job exposure matrix (JEM) [41,42].

The study conducted by Blanc-Lapierre et al. in 2017 [36] in Canada found a weak association between perceived stress at work and PCa, with an odds ratio (OR) of 1.26 (95% confidence interval (CI): 0.95–1.68). Their later study in 2017 [37], with a larger sample size, reinforced the same finding, with an OR of 1.12 (95% CI: 1.04–1.20), indicating a slightly increased risk of PCa with work stress before age 65. In 2018, the same group [38] reported that exposure to benzene, toluene, xylene (BTX), and styrene, common substances in various industrial processes, was associated with increased PCa risk (OR for BTX: 1.27; 95% CI: 1.05–1.35; OR for styrene: 1.19, 95% CI: 0.74–1.91).

In an Australian study by Doolan et al. (2014) [39], physical activity at work was evaluated, showing a marginally increased risk (OR: 1.15; 95% CI: 0.95–1.40). Meanwhile, studies in the USA and Canada focusing on firefighters [40,44] observed a more significant increase in PCa risk related to firefighting activities, with ORs of 1.36 (95% CI: 1.27–1.46) and 1.45 (95% CI: 1.25–1.69), respectively.

Two separate Spanish studies [25,41] exploring the impact of night shift work (NSW) and exposure to alkylphenolic compounds, respectively, reported ORs close to 1, indicating no significant increase in PCa risk. However, another study conducted in France by Wendeu-Foyet et al. (2018) [45] showed a more pronounced association between NSW and PCa, especially for long hours and consecutive nights.

Another interesting finding was reported in a Canadian study by Peters et al. (2016) [42], suggesting that solar ultraviolet radiation, a risk factor for skin cancer, could potentially lower the risk of PCa (OR: 0.68, 95% CI: 0.51–0.92) in outdoor workers.

A comprehensive study by Sharma et al. (2015) [43] explored several risk factors associated with farming jobs, including residence and pesticide use. The study revealed variable risk levels depending on the specific exposure, with farm residence and combined pesticide use showing higher ORs.

## 4. Discussion

In this systematic review, we investigated a collection of 11 studies from various geographical locations exploring the relationship between different occupational exposures and the risk of prostate cancer. The presented studies demonstrate the potential for various occupational exposures to influence PCa risk. Several studies focused on perceived stress at work; physical activity at work; and exposure to specific substances such as benzene, toluene, xylene (BTX), styrene, and alkylphenolic compounds. Others evaluated more specific occupational contexts, such as firefighting, farming, and night shift work or environmental exposure, like solar ultraviolet radiation. The outcome measures used to quantify the association between exposure and PCa also varied, most commonly employing a combination of questionnaires, interviews, occupational classifications, registries, and job exposure matrices (JEMs).

The review found a diverse range of associations between occupational exposure and PCa risk, supporting previous research suggesting that the work environment can have profound implications for employee health, especially with respect to chronic conditions like prostate cancer.

The studies conducted by Blanc-Lapierre et al. in 2017 highlighted perceived work stress as a potential risk factor for PCa [36,37]. This aligns with previous research linking chronic psychological stress to various health complications, including the initiation and progression of multiple cancer types. Such mechanisms might involve stress-induced changes in gene expression or immunosuppression, which may promote cancer development and progression [46,47,48,49].

Blanc-Lapierre et al. (2018) investigated the impact of exposure to benzene, toluene, xylene (BTX), and styrene [38]. In this particular study, BTX exposure was found to have a significant association with an increased risk of prostate cancer, reinforcing previous findings that underscored the potential carcinogenic effects of these substances. Long-term exposure to BTX, especially in high concentrations, has been linked to various health complications, including hematological malignancies and other cancers [50,51]. Extending this risk to PCa signifies an important direction for future occupational health research.

On the other hand, our study found no significant association between exposure to styrene and the risk of prostate cancer. This is particularly intriguing, given that styrene has been classified as a possible human carcinogen and is known to cause a range of health problems upon long-term exposure [52]. However, the effect of styrene exposure on the risk of PCa specifically remains ambiguous and requires further investigation. The findings reported by Blanc-Lapierre et al. (2018) contribute to this ongoing discourse, suggesting that the relationship might be more complex than initially anticipated [38].

The risk posed by firefighting as an occupation, as investigated in studies by Lee et al. (2020) and Tsai et al. (2015), has been previously documented [40,44]. Firefighters are exposed to a variety of carcinogens during the course of their duties, which could explain the elevated incidence of not only prostate cancer but also other types of cancer. This reinforces the importance of protective measures, including appropriate use of personal protective equipment and hygiene practices, to reduce exposure [28,33,53,54].

Interestingly, a study by Peters et al. (2016) found an inverse relationship between outdoor workers’ exposure to solar ultraviolet radiation and PCa [42]. This could potentially be explained by the protective role of vitamin D synthesized in skin upon exposure to ultraviolet-B radiation. Vitamin D has been implicated in the regulation of cell growth and immune function, potentially offering some protection against cancer [55,56,57,58].

According to Doolan et al. (2014) [39], physical activity at work was associated with PCa risk, with modestly increasing odds ratios, although the association was non-significant. While exercise is generally seen as protective against numerous health issues, the specific nature and conditions of physical work may lead to different effects, possibly related to stress and exhaustion factors [59,60].

Sharma et al. (2015) presented a complex set of findings within the farming context [43]. While residing on a farm was found to be associated with an elevated PCa risk, the role of exposure to specific agricultural chemicals, such as insecticides and fungicides, was less clear. Some types of pesticides were associated with increased risk, while others showed no significant effect. These results might be reflective of the heterogeneous nature of agricultural practices and exposures and underline the need for more specific investigations into the role of individual pesticides and other farming-related exposures in PCa development [61,62,63,64,65,66].

Moreover, night shift work, with its inherent disruption of circadian rhythms, has been hypothesized to contribute to cancer risk. The studies by Papantoniou et al. (2015) and Wendeu-Foyet et al. (2018) provide some support for this theory, especially among those with long-term and heavy exposure to night work [25,45]. Night work, particularly when it involves frequent or extended hours, can disrupt circadian rhythms and other biological processes, which, in turn, can have a variety of health impacts.

Circadian disruption due to night shift work has been hypothesized to contribute to increased cancer risk due to several potential mechanisms, including the suppression of melatonin production, which normally occurs at night. Melatonin is an endogenous hormone known for its antioxidant properties and its role in regulating the immune system, among other functions. Reduced melatonin levels have been associated with an increased risk of several types of cancer, including PCa [67,68,69,70,71]. A perturbative effect of shift work on testosterone serum levels was evidenced in a sample of male night shift workers [72]. Moreover, shift workers were found to have significantly lower levels of vitamin D [73].

Circadian disruption can also lead to sleep deprivation, which has been associated with inflammation, immune suppression, and other physiological changes that can potentially increase cancer risk [74,75,76].

Importantly, the study by Wendeu-Foyet et al. (2018) suggests that the duration and intensity of night work exposure may play a critical role in influencing PCa risk [45]. This implies that all night shift work may not carry the same level of risk and that the specific conditions of work (e.g., the frequency of night shifts, the number of consecutive night shifts, and the duration of exposure over a lifetime) can moderate this association.

However, as the mechanisms linking night shift work and cancer are still not fully understood, further research is needed to corroborate these findings and elucidate the biological pathways involved. Moreover, future studies should consider potential confounding factors, such as lifestyle behaviors or other occupational exposures, that could influence the observed associations [72,77,78,79,80].

The study by Peremiquel-Trillas et al. (2019) broadens our perspective to the industrial environment, looking at exposure to alkylphenolic compounds [41]. While these substances have endocrine-disrupting properties and therefore could theoretically contribute to PCa risk, the study did not find a significant association. This underlines the complexities of investigating associations with endocrine disruptors, which can depend on a multitude of factors, including timing, dosage, and individual susceptibility [81,82,83].

The findings of this systematic review, encompassing eleven studies, underscore the important implications of occupational exposures for PCa risk. The potential risks associated with specific chemical substances, such as BTX [38] and alkylphenolic compounds [41], as well as the links of physical activity at work [39], stress at work [36,37], and night shift work [25,45] with prostate cancer, offer new avenues for prevention and control strategies in occupational health.

For instance, the findings related to chemical exposures might encourage the implementation of better industrial hygiene practices and the development of safer alternatives to hazardous substances. Furthermore, the research on night shift work suggests the importance of maintaining healthy circadian rhythms, possibly by incorporating regular breaks and rotating schedules for shift workers.

The results also demonstrate the necessity for broader public health interventions. For instance, the significant findings related to firefighting activities [40,44] and farming practices [43] may imply the need for improved protective measures and regulations within these professions, including enhanced protective equipment and regular health screenings. It is well-established that early detection through PSA screenings can lead to better prognosis and outcomes for PCa patients [84]. Given the evidence suggesting increased risk of PCa in certain occupational groups, it seems prudent to consider enhanced surveillance strategies for these groups. Tailored screening, possibly starting at a younger age or with more frequent intervals, could be beneficial for individuals with prolonged exposure to known occupational risk factors. For instance, firefighters, who are repeatedly exposed to carcinogens during their service, might benefit from such a tailored approach [85]. Similarly, those involved in jobs with consistent exposure to chemicals like benzene or toluene could also be considered for enhanced surveillance [38]. However, it is essential that any recommendations for altered screening strategies be made in alignment with the broader clinical and epidemiological evidence and in consideration of potential overdiagnosis or overtreatment [86,87,88].

The studies under review underscore the fact that the relationship between occupational exposure and the risk of developing PCa is multifaceted, often interacting with other factors. For instance, age, ancestry, family history of prostate cancer, education, income, marital status, body mass index, type 2 diabetes, alcohol consumption, smoking, physical activity, and dietary habits have all been controlled for in various studies, highlighting their potential role in modulating the effect of occupational exposure on PCa risk [89,90].

Ancestry and family history of prostate cancer, in particular, are well-documented risk factors for prostate cancer, and their interaction with occupational exposure might influence individual susceptibility to this disease [91].

Body mass index (BMI) and type 2 diabetes, which are indicative of overall metabolic health, may also interact with occupational risk factors. Obesity is associated with chronic low-grade inflammation and hormonal changes, which may amplify the carcinogenic effects of certain occupational exposures [92,93]. Similarly, type 2 diabetes may increase PCa risk by influencing insulin and insulin-like growth factor pathways, potentially enhancing the carcinogenic impact of certain workplace exposures [94].

Lifestyle factors such as smoking and alcohol consumption, which can independently increase the risk of various cancers, might also interact with occupational exposure. For instance, they may contribute to a higher burden of overall oxidative stress and DNA damage, increasing the likelihood of carcinogenic transformations in the presence of occupational hazards [95,96].

Physical activity, either at work or during leisure time, and dietary habits, especially the intake of fruits and vegetables, may also modulate PCa risk; hence, their interaction with occupational exposure warrants further research [97,98,99,100].

Exposure to specific carcinogens, such as BTX, styrene, and alkylphenolic compounds, among others, may interact synergistically or additively with other factors, increasing the overall risk of PCa [101,102].

Moreover, understanding the mechanisms through which occupational exposures contribute to prostate cancer is fundamental to clarify the etiological pathways. First, firefighting exposures often involve contact with carcinogenic substances like PAHs.

PAHs are known to form DNA adducts, leading to mutations that may drive carcinogenesis in the prostate [103]. Moreover, firefighters are exposed to other carcinogens such as formaldehyde and acrolein, which can cause DNA crosslinking and contribute to genomic instability [104].

Physical activity has predominantly been associated with protective effects against various cancers due to its anti-inflammatory effects, improved insulin sensitivity, and modulation of sex hormone levels [105]. However, excessive physical activity might lead to hormonal imbalances, particularly in testosterone levels, which are implicated in prostate cancer progression [106].

Night shift work, with its associated circadian disruption, affects melatonin secretion. Melatonin is a potent antioxidant that also modulates the immune response and apoptosis [107]. Circadian disruption may lead to increased oxidative stress and immunosuppression, providing a favorable environment for prostate tumorigenesis [108].

Occupational exposure to chemicals like benzene, toluene, and xylene can impact various cellular processes. Benzene metabolites can induce oxidative stress, causing DNA strand breaks and chromosomal aberrations [109]. Chronic exposure to these chemicals may also cause epigenetic alterations, leading to abnormal gene expression patterns associated with prostate cancer [110].

Solar ultraviolet (UV) radiation indirectly affects prostate cancer through vitamin D synthesis. Vitamin D exerts its effects through the vitamin D receptor (VDR), which is involved in cell differentiation, proliferation, and apoptosis [111]. Altered VDR expression or vitamin D deficiency may contribute to prostate cancer progression by disrupting these cellular processes [112].

These molecular alterations collectively provide insight into the potential mechanisms linking occupational exposures to prostate cancer risk.

In relation to the potential biomarkers linked to occupational exposures and prostate cancer, some biomarkers of exposure should be used.

For instance, firefighters’ exposure to PAHs can lead to the formation of PAH-DNA adducts, serving as biomarkers for genotoxic exposure [103]. Workers exposed to chemical agents might display elevated levels of urinary benzene metabolites, which are indicative of organic solvent exposure, which could indirectly point to prostate cancer susceptibility [113]. Additionally, night shift workers experiencing circadian rhythm disruptions often present with altered melatonin levels, which have been postulated as a potential biomarker for various cancers, including prostate cancer [114]. For those exposed to UV radiation, serum vitamin D levels might act as a potential biomarker, considering the pivotal role of vitamin D in cellular processes and its association with prostate cancer [111].

An inherent limitation of our review is the variability in exposure classifications across the referenced studies. Some studies provided specific thresholds or criteria for classifying exposure, while others adopted a broader definition. This inconsistency can introduce potential uncertainties in interpreting the observed associations, especially when comparing results across different studies.

In light of the findings, it is abundantly clear that investing in the four Ps of medicine—predictive, preventive, personalized, and participatory—could significantly contribute to addressing the multifaceted issue of PCa risk in various occupations by harnessing the protective factors and mitigating the risk factors identified in the studies.

Moreover, a cumulative risk assessment approach that considers the combined effect of multiple risk factors rather than their individual impact can likely provide a more accurate measure of PCa risk. This shift in perspective from a single risk factor to a cumulative risk approach would not only improve our understanding of PCa epidemiology but also enhance our ability to design and implement effective prevention strategies.

Furthermore, the adoption of Total Worker Health^®^ approaches aimed at improving the well-being of workers by eliminating modifiable risk factors could prove invaluable. This includes reducing exposure to harmful substances, promoting healthy habits like regular physical activity and a balanced diet, and ensuring regular health screenings.

Lastly, the findings emphasize the importance of continued epidemiological research to elucidate the relationships between various occupational exposures and PCa risk. Improved understanding of these relationships can inform preventive measures, aid in early detection efforts, and contribute to more effective treatments, thereby reducing the burden of prostate cancer.

## 5. Conclusions

This systematic review underscores the complex interplay of occupational exposures and other risk factors in the etiology of PCa. It is clear from the gathered evidence that certain occupational exposures, such as firefighting; physical activity; night shift work; and exposure to alkylphenolic compounds, solar ultraviolet radiation, and certain chemicals, can significantly influence the risk of developing prostate cancer. Importantly, the risk conferred by these factors can be further modulated by various individual and lifestyle factors, necessitating a comprehensive approach to risk assessment.

The evidence from these studies strongly advocates for the adoption of the four Ps of medicine, i.e., predictive, preventive, personalized, and participatory healthcare strategies.

Moreover, the review emphasizes the necessity of adopting a cumulative risk assessment approach, taking into account the combined effects of multiple risk factors rather than their individual contributions. Our review also highlights the importance of a Total Worker Health^®^ approach with the aim of improving worker health by eliminating modifiable risk factors and promoting overall well-being.

Despite the significant strides made in understanding the occupational risk factors for PCa, there remain gaps in our knowledge, especially concerning the nuanced molecular mechanisms at play and the exact role of potential biomarkers in signaling exposure-related risks. Further studies are needed to delve deeper into exposure duration, intensity, and latency periods, which might modulate PCa risk. There is also a significant need to study synergistic interactions among various occupational exposures and to evaluate if certain subpopulations are more genetically predisposed to PCa when subjected to specific occupational risks.

Finally, further research is required to fully elucidate these relationships and the mechanisms underlying them. We hope that continued advancements in this area will pave the way for the development of more effective strategies for preventing and managing PCa, ultimately improving the lives of workers worldwide.

## Figures and Tables

**Table 1:** Studies evaluating association between occupational exposure and prostate cancer.

Author (Year)	Country	Study Size		Occupation	Exposure	Assessment Technique	Adjustment Level	Effect Estimate OR 95%: CI Inf–CI Sup
		Cases	Control					
Blanc-Lapierre et al. (2017)a [36]	Canada	328	400	Various	Perceived stress at work	Questionnaire and interviews	Age, ethnicity, education level, family income, respondent status, and site-specific non-occupational and occupational covariates	1.26 (0.95–1.68)
Blanc-Lapierre et al. (2017)b [37]	Canada	1.933	1.994	Various	Perceived stress at work before age 65	Questionnaire and interviews	Age, ancestry, first-degree family history of PCa, family income, education, marital status, body mass index, type 2 diabetes, depression treated with medication, alcohol consumption, smoking, physical activity at work, and frequency of fruit and vegetable intake	1.12 (1.04–1.20)
Blanc-Lapierre et al. (2018) [38]	Canada	1920	1989	Various	Benzene, toluene, xylene (BTX), and styrene	Canadian occupational classification	Age, ancestry, first-degree family history of PCa, household income, education, body mass index, type 2 diabetes, alcohol consumption, smoking, and physical activity at work	BTX 1.27 (1.05–1.35) Styrene 1.19 (0.74–1.91)
Doolan et al. (2014) [39]	Australia	1.436	1.436	Various	Physical activity at work	Questionnaire	Age, family history, and SEIFA index of economic resources	1.15 (0.95–1.40)
Lee et al. (2020) [40]	U.S.A.	1.119	63.912	Firefighters	Firefighting	Firefighter registry	Age at cancer diagnosis	1.36 (1.27–1.46)
Papantoniou et al. (2015) [25]	Spain	1.095	1.388	Various	Night shift work (NSW)	Nested data from the MCC-Spain study	Age, center, educational level, family history of prostate cancer, physical activity over the past decade, smoking status, past sun exposure, and daily meat consumption	Ever NSW 1.14 (0.94–1.37) Permanent NSW 1.10 (0.85–1.43) Rotating NSW 1.16 (0.95–1.46)
Peremiquel-Trillas et al. (2019) [41]	Spain	1.095	1.480	Various	Alkylphenolic compounds	Job exposure matrix (JEM)	Age, region, education level, BMI, smoking, alcohol consumption, occupational shift, exposure to pesticides, and exposure to solvents	1.09 (0.89–1.33)
Peters et al. (2016) [42]	Canada	1.638	1.697	Outdoor workers	Solar ultraviolet radiation	Job exposure matrix (JEM)	Relationship status, smoking, education, and fruit and vegetable consumption	0.68 (0.51–0.92)
Sharma et al. (2015) [43]	Canada	114	2.824	Farming job	Farm risk	Questionnarie	Residence and family history of cancer	Farming job 1.43 (0.70–2.92) Farm residence 1.86 (1.07–3.25) Insecticide 1.31 (0.55–3.15) Fungicides 0.98 (0.26–3.63) Both pesticides 2.23 (1.15–4.33) Radiation 1.97 (1.04–3.74)
Tsai et al. (2015) [44]	U.S.A.	1.397	48.825	Firefighters	Firefighting	Firefighter registry	Age at cancer diagnosis	1.45 (1.25–1.69)
Wendeu-Foyet et al. (2018) [45]	France	818	875	Various	Night shift work (NSW)	Questionnaire and interviews	Age, family history of prostate cancer, race, and education level	Evening chronotype 1.83 (1.05–3.19) 20 yr permanent NSW 1.76 (1.13–2.75) >10 h NSW 4.64 (1.78–12.13) ≥6 consecutive nights 2.43 (1.32–4.47)

## Data Availability

Data sharing not applicable.
